# Genomic and Phenotypic Characterization of Shiga Toxin-Producing *Escherichia albertii* Strains Isolated from Wild Birds in a Major Agricultural Region in California

**DOI:** 10.3390/microorganisms11112803

**Published:** 2023-11-18

**Authors:** Michelle Qiu Carter, Beatriz Quiñones, Xiaohua He, Antares Pham, Diana Carychao, Michael B. Cooley, Chien-Chi Lo, Patrick S. G. Chain, Rebecca L. Lindsey, James L. Bono

**Affiliations:** 1 Produce Safety and Microbiology Research Unit, U.S. Department of Agriculture, Western Regional Research Center, 800 Buchanan Street, Albany, CA 94710, USA; beatriz.quinones@usda.gov (B.Q.); apdienc@gmail.com (A.P.); diana.carychao@usda.gov (D.C.); masdpcooley@gmail.com (M.B.C.); 2 Foodborne Toxin Detection and Prevention Research Unit, U.S. Department of Agriculture, Western Regional Research Center, Albany, CA 94710, USA; xiaohua.he@usda.gov; 3 Biosecurity and Public Health Group, U.S. Department of Energy, Los Alamos National Laboratory, Santa Fe, NM 87545, USA; chienchi@lanl.gov (C.-C.L.); pchain@lanl.gov (P.S.G.C.); 4 Enteric Diseases Laboratory Branch, Centers for Disease Control and Prevention, Atlanta, GA 30329, USA; wmi1@cdc.gov; 5 Meat Safety and Quality Research Unit, U.S. Department of Agriculture, U.S. Meat Animal Research Center, Clay Center, NE 68933, USA; jim.bono@usda.gov

**Keywords:** *Escherichia albertii*, pangenome, Shiga toxin, cytolethal distending toxin, intimin, cytotoxicity, pathogenicity, phage integration, *stx*_2f_, foodborne pathogen

## Abstract

*Escherichia albertii* is an emerging foodborne pathogen. To better understand the pathogenesis and health risk of this pathogen, comparative genomics and phenotypic characterization were applied to assess the pathogenicity potential of *E. albertii* strains isolated from wild birds in a major agricultural region in California. Shiga toxin genes *stx*_2f_ were present in all avian strains. Pangenome analyses of 20 complete genomes revealed a total of 11,249 genes, of which nearly 80% were accessory genes. Both core gene-based phylogenetic and accessory gene-based relatedness analyses consistently grouped the three *stx*_2f_-positive clinical strains with the five avian strains carrying ST7971. Among the three Stx2f-converting prophage integration sites identified, *ssrA* was the most common one. Besides the locus of enterocyte effacement and type three secretion system, the high pathogenicity island, OI-122, and type six secretion systems were identified. Substantial strain variation in virulence gene repertoire, Shiga toxin production, and cytotoxicity were revealed. Six avian strains exhibited significantly higher cytotoxicity than that of *stx*_2f_-positive *E. coli*, and three of them exhibited a comparable level of cytotoxicity with that of enterohemorrhagic *E. coli* outbreak strains, suggesting that wild birds could serve as a reservoir of *E. albertii* strains with great potential to cause severe diseases in humans.

## 1. Introduction

*Escherichia albertii*, an emerging human and avian pathogen, was first isolated from Bangladeshi children with diarrhea in 1991 and classified initially as *Hafnia alveri* [1]. In 2003, these isolates were reclassified as *E. albertii* based on their biochemical properties, DNA hybridizations, and 16S rRNA gene sequences [2]. *E. albertii* is the most divergent lineage in the genus *Escherichia* and is closely related to *Shigella boydii* based on the extended multilocus sequence typing (MLST) and phylogenetic analyses [3,4]. As an avian pathogen, *E. albertii* was the cause of the large-scale mortality of redpoll finches (*Carduelis Flammea*) in Alaska and contributed to several mortality incidents of wild finches (*Fringillidae*) in Scotland [5,6]. In humans, *E. albertii* causes diarrhea, abdominal pain, and high fever, although bacteremia and extraintestinal infections were also reported [7,8]. Sporadic outbreaks of *E. albertii* infections have been reported worldwide, particularly in Japan [9,10,11,12,13,14]. Transmission of *E. albertii* is thought to occur mainly via contaminated food or water. Although *E. albertii* has been isolated from various domestic and wild animals, poultry and wild birds are considered the primary reservoirs [15]. 

One of the characteristics of *E. albertii* pathogenesis is the formation of attaching-and-effacing (A/E) lesions on the surfaces of intestinal epithelial cells, similar to other attaching-and-effacing pathogens, including enteropathogenic *E. coli* (EPEC), enterohemorrhagic *E. coli* (EHEC), and *Citrobacter rodentium* [16]. Similarly, genes responsible for formation of A/E lesions in *E. albertii* are located on a locus of enterocyte effacement (LEE) island, including the intimin gene *eae* and the gene (*tir*) encoding the translocated intimin receptor protein. Additionally, LEE in *E. albertii* carries the genes involved in the biogenesis of the type three secretion system (T3SS) apparatus and several T3SS effector genes [17]. Interestingly, some *E. albertii* strains harbor a genomic island (GI) encoding a second T3SS (ETT2), a cryptic T3SS distantly related to the *Salmonella* T3SS [18]. The function of ETT2 was originally thought to encode a secretion system for delivery of effectors to host cells, as it carries genes encoding T3SS components. However, recent studies demonstrated that rather than secretion of effectors, ETT2 is involved in the secretion of flagellar proteins, fimbrial proteins, and cell surface proteins and contributes to bacterial motility and resistance to detergents and hydrophobic antibiotics [19]. The genes *cdtABC,* encoding the cytolethal distending toxin (CDT), are conserved in *E. albertii*. CDT is composed of three subunits. The CdtA and CdtC subunits function together to mediate the binding of the holotoxin to eukaryotic cells and the subsequent delivery of CdtB into the host cell. CdtB possesses DNase I activity and induces host cell cycle arrest and, eventually, apoptosis via DNA damage [20,21]. Shiga toxin genes have been detected in a subset of *E. albertii* strains, in which subtype *stx*_2f_ is common, with one exception of an *stx*_2a_-positive clinical isolate in Norway [22]. Possession of both *eae* and *stx*_2a_ may increase the risk of developing hemolytic uremic syndrome (HUS) during *E. albertii* infections, a clinical signature of EHEC infections [23]. Other reported virulence genes include *paa*, encoding the porcine attaching-effacing-associated protein that plays a role in the pathogenesis of EPEC and enterotoxigenic *E. coli* (ETEC) [24].

Recently, *E. albertii* strains isolated from infected humans, environmental samples, poultry, and healthy and diseased wild birds have been reported worldwide [9,12,25,26]. Genomic characterization is desired, as such a study will provide a comprehensive picture of virulence gene repertoire as well as uncover valuable information about disease ecology and public health associated with this pathogen. Additionally, considering the long-distance migration of wild birds, a deeper understanding of the role of the avian reservoirs in *E. albertii*’s dispersal will provide valuable information for the development of effective measures for controlling the transmission of this human pathogen. In our previous study that assessed the prevalence of Shiga toxin-producing *E. coli* (STEC) in a leafy greens-growing region in Central California [27], *E. albertii* strains were isolated from many wild birds near this agricultural area. In this study, we performed comparative genomics and pathogenomic analyses of ten wild bird *E. albertii* strains with a set of *E. albertii* clinical strains isolated from 1954–2014 in the United States. We further performed comparative phenotypic analyses with several STEC and EHEC strains to determine Stx production and Vero cell-based cytotoxicity. Our study uncovered novel virulence determinants in avian and clinical *E. albertii* strains and revealed that wild birds, especially American crow (*Corvus brachyrhynchos*) and brown-headed cowbird (*Molothrus ater*), carried Shiga toxin-producing *E. albertii* (STEA) strains that have an increased potential to cause severe diseases in humans. 

## 2. Materials and Methods

### 2.1. Bacterial Strains and Growth Media 

*E. coli* and *E. albertii* strains and their sources are listed in Table 1. Environmental strains were isolated by cloacal swab from captured birds near a major agricultural region in California, as described previously [28]. Wildlife sampling at all locations was approved under a set of California Department of Fish and Game (CDFG) Scientific Collection Permits issued to USDA Wildlife Services and CDFG personnel contracted to collect the samples and ship to USDA in Albany, California. The wildlife sampling was conducted through a contract with state and federal wildlife agencies using their standard protocols. The strains were grown routinely in Luria-Bertani (LB) broth (10 g tryptone, 5 g yeast extract, and 5 g NaCl per liter). 

### 2.2. Genome Sequencing and Annotation 

The genomes of the ten avian *E. albertii* strains were sequenced using an RS II instrument (Pacific Biosciences, Menlo Park, CA, USA) as previously reported [36]. Briefly, bacterial DNA was extracted from the mid-exponential phase cultures grown in LB broth and resuspended in Qiagen Elution Buffer (QIAGEN, Germantown, MD, USA) for genome sequencing. Genomic libraries were prepared according to the PacBio (Pacific Biosciences) 20 Kb library standard protocol, and DNA sequencing was performed with P6/C4 sequencing chemistry and 360 min data collection protocol. The sequence reads were filtered with a PreAssembler Filter prior to de novo assembly with RS_HGAP_Assembly v.3. 

To obtain complete genome sequences of the two clinical strains that only have draft genome sequences (Table 1), bacterial DNA was extracted from exponential phase cultures grown in LB broth as previously reported [37]. DNA (10 µg) was sheared to a 30 Kb target fragment length using g-TUBEs (Covaris, Woburn, MA, USA) and concentrated with 0.45× volume AMPure PB beads (Pacific Biosciences). Sheared and concentrated DNA (5 µg) was used to make PacBio sequencing libraries using the SMRTbell Prep Kit 3.0 and barcoded using the SMRTbell barcoded adapter plate 3.0. The Sequel II binding kit 3.2 and Sequel II sequencing plate 2.0 were used to run the library on a Sequel IIe system with the application HiFi reads and a 30 h movie time with a 6 h pre-extension. PacBio reads were assembled using Microbial Genome Analysis in SMRT analysis v 10.1 and contigs imported into Geneious Prime^®^ (Dotmatics, Bishop Stortford, UK or Boston, MA, USA). The overlapping sequences on the ends of the contigs were removed from the 5′ and 3′ ends to generate circularized chromosomes and plasmids. Closed chromosomes were reoriented using Ori-Finder 2 [38], and all subsequent genomes were oriented to the same start position. The closed chromosome and plasmids were manually polished by mapping Illumina and PacBio reads to the chromosome and known plasmids using Geneious mapper. Unused reads were de novo assembled using the Geneious assembler for small plasmid identification. All 12 complete genome sequences were submitted to GenBank and annotated with the NCBI Prokaryotic Genome Annotation Pipeline [39] (Table 1). 

### 2.3. Pangenome Analyses 

The pangenome of *E. albertii* was calculated using Roary (V3.13.0) as previously reported [40]. Briefly, the annotated Coding DNA Sequences (CDSs) were extracted from each genome and converted to protein sequences. To identify homologous proteins, all-against-all comparison was performed using BLASTP with 95% as the minimum percentage identity. The resulting core genes were aligned using MAFFT (V7.471), and a maximum-likelihood tree was constructed in IQ-TREE (version 2.1.2) using the best-fit model GTR+F+I, as selected by ModelFinder [41,42]. To identify strains carrying more common genes, an approximate-maximum-likelihood tree of the binary accessory genes was constructed in FastTree (v2.1.10) with the default settings (Jukes-Cantor + CAT model or GTR+CAT model) as described previously [40]. The pangenome-wide association analysis was performed using Scoary (v1.6.16) to reveal host-associated genes and/or strain-specific genes. 

### 2.4. Identification of Virulence Genes and Pathogenicity Islands (PAIs) 

*E. coli* virulence genes listed in the Virulence Factor DataBase (VFDB) as of May 2023 were used as queries of BLASTn to search the genomes examined in this study (Table 1). The BLASTn was performed in Geneious Prime^®^ with a threshold of 65% for gene coverage and 70% or 25% for sequence identity at the nucleotide or amino acid level, respectively. BLASTn searches were first performed to identify homologs of a genomic island (GI) or a PAI in all genomes using Geneious Prime^®^ as previously reported [40,43]. When a complete GI or PAI was not detected, all CDSs encoded by the query GI or PAI were used to search the genome of the testing strain by BLASTP. The association of the T3SS effector genes and toxin genes was assessed by first calculating the median difference for each examined gene between the clinical and wild bird strains. Following this initial comparison, a list of genes that were different between the two isolation sources by greater than two standard deviations were subsequently selected and evaluated with the Fisher’s exact test with the Bonferroni correction for multiple comparison using R version 4.3.0. as detailed in previous reports [44,45].

### 2.5. Comparative Analysis of Stx-Prophages 

The complete genome sequence of each strain was submitted to PHASTER [46] for identification of prophage and prophage-like elements. The putative integration sites were initially identified by PHASTER and confirmed in Geneious Prime^®^ using “Find Repeats” in the defined chromosomal regions. The Stx-prophage genomes were extracted from the corresponding bacterial genomes. The Stx-prophage genomes were aligned using Clustal Omega in Geneious Prime^®^. A neighbor-joining consensus tree was constructed with the following parameters: Genetic Distance Model, Jukes-Cantor; Resampling Method, bootstrap; and number of replicates, 10,000. 

### 2.6. Quantification of Shiga Toxin by ELISA 

The amount of Stx in each bacterial supernatant was quantified by ELISA, as described previously [47]. Briefly, a 96-well plate was coated at 4 °C overnight with a monoclonal antibody against Stx2 at a concentration of 5 µg/mL, followed by incubation with 3% BSA at 37 °C for 1 h. The Stx2f was detected using biotinylated mouse monoclonal antibody Stx2f-4 [48] in conjugation with streptavidin-HRP at a concentration of 0.2 µg/mL (Invitrogen, Waltham, MA, USA). The plates were developed using SuperSignal West Pico Chemiluminescent Substrate (Thermo Scientific, Waltham, MA, USA), and the luminescence was measured using the Victor-3 plate reader (Perkin-Elmer, Shelton, CT, USA). The concentration of Stx2f in each sample was calculated using the standard curves generated with purified Stx2f. 

### 2.7. Cytotoxicity Assay 

The Stx activity from each strain was determined with a mammalian Vero cell line, Vero-d2EGFP with a destabilized variant (t1/2 = 2 h) of the enhanced green fluorescent protein (EGFP) [49]. Briefly, Vero-d2EGFP cell intoxications were performed after incubation at 37 °C in a 5% CO_2_ humidified incubator with Ham’s F-12 Nutrient medium (Life Technologies) containing tenfold dilutions of cell-free culture supernatants from each bacterial strain. After 20 h, the Vero-d2EGFP cells were briefly rinsed with 1X phosphate-buffered saline, and the EGFP fluorescence was measured using a BioTek Synergy HT Multi-Detection Microplate Reader (Agilent Technologies, Santa Clara, CA, USA), as described in a previous study [49]. Statistical analysis of the cytotoxicity levels among the tested strains was performed with R software version 4.3.0 (R Core Team 2022). 

## 3. Results

### 3.1. Pangenome of E. albertii 

**Figure 1:**
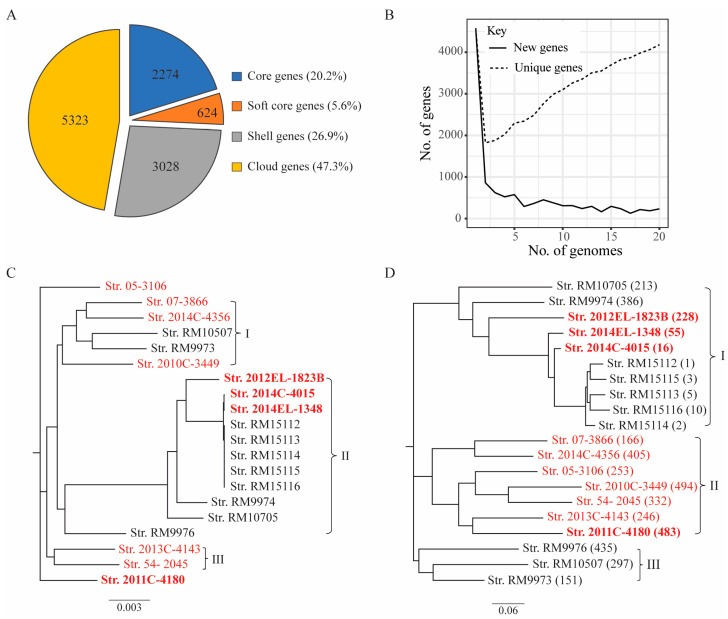
Pangenome of *E. albertii* strains. (**A**) Core and accessory genes of *E. albertii*. The numbers of genes in the core and accessory genomes were calculated using Roary (V3.13.0), as detailed in the Materials and Methods section. The core genes refer to all genes shared by all input genomes (*n* = 20); the soft-core genes refer to all genes present in all 19 input genomes; the shell genes refer to all genes present in at least 3 but less than 19 input genomes; the cloud genes refer to all genes present in less than 3 input genomes. (**B**) Change in the total number of new genes and unique genes as genomes are added in random orders. As the number of genomes used for the pangenome analyses increased, the total number of new genes (genes not found in the previously compared genomes) decreased, while the total number of unique genes (genes unique to individual strains) increased. (**C**) Core genome-based phylogeny of the *E. albertii* strains. The Roary matrix contained a total of 11,249 gene clusters. Strain names in red are clinical strains, and strain names in red bold are *stx*_2f_-positive clinical isolates. (**D**) The accessory genome-based strain relatedness. The number in the parentheses represents the number of strain-specific genes. Strain names in red are clinical strains, and strain names in red bold are *stx*_2f_-positive clinical strains.

Pangenome analyses of the 20 *E. albertii* strains revealed a total of 11,249 genes, among which 2274 genes were present in all genomes (core genes, 20.2%) (Figure 1A). The accessory genes included 3028 genes present in more than 3 but less than 19 genomes (shell genes, 26.9%) and 5325 genes present in less than 3 genomes (cloud genes, 47.3%), implying that *E. albertii* carried a large number of dispensable genes. This large accessory genome showed the great genetic diversity in this species, which was further supported by the observation that the number of unique genes increased when the number of genomes increased (Figure 1B). Core gene-based phylogeny analysis placed the strains in three clades and two singletons but failed to separate clinical strains from the non-clinical strains (Figure 1C). The largest clade (Clade II) contained three *stx*_2f_-positive clinical strains and eight wild bird strains. The other *stx*_2f_-positive clinical strain (2011C-4180) formed a singleton. The second-largest clade contained two *stx*-negative clinical strains, the chicken carcass strain (*stx*-negative), and two wild bird strains that both harbored the *stx*_2f_ genes. The relatedness of clinical and avian strains was further evaluated with a tree constructed based on the presence of the accessory genes (Figure 1D). Three clades were revealed, among which the largest one (Clade I) contained the three *stx*_2f_-positive clinical strains and seven wild bird strains. The second clade contained all other clinical strains and the chicken carcass strain, while the third clade contained the rest of the three wild bird strains. Therefore, the three *stx*_2f_-positive clinical strains, 2012EL-1823B, 2014C-4015, and 2014EL-1348, appeared to be phylogenetically highly related to a clonal group of wild bird strains belonging to ST7971 (RM15112-RM15116) and also shared more accessory genes with this group of STEA strains than any other STEA strains examined in this study (Table 1).

### 3.2. Analyses of PAIs in E. albertii 

Seven PAIs, including the high pathogenicity island (HPI), locus of adhesion and autoaggregation (LAA), LEE, locus of proteolysis activity (LPA), OI-122, OI-57, and the subtilase-encoding pathogenicity island (SE-PAI) as described previously [40,43], and the gene clusters encoding the type six secretion system (T6SS) were examined in detail in *E. albertii* strains. Complete or partial homologs of LEE, OI-122, HPI, and T6SS were detected in *E. albertii*, while homologs for others were not identified.

#### 3.2.1. LEE 

**Figure 2:**
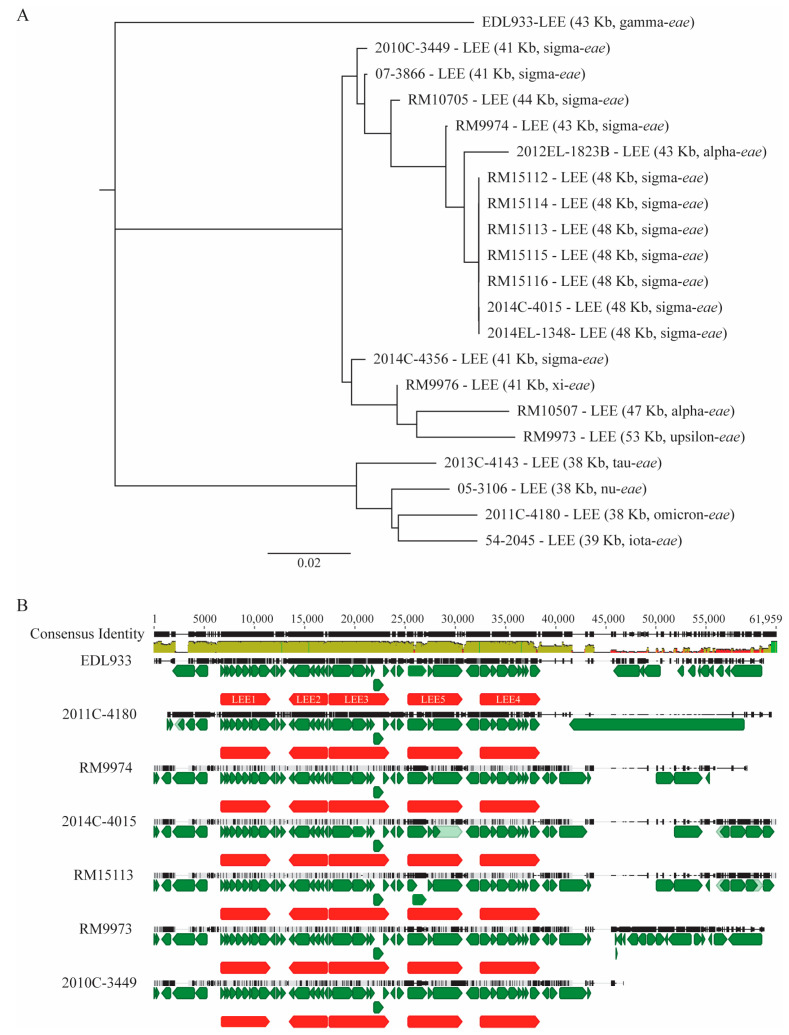
Sequence analyses of LEE in *E. albertii*. (**A**) Phylogeny of *E. albertii* LEEs. The LEEs in the *E. albertii* strains were identified by BLASTn search of a database containing all genomes examined in this study using DNA sequence of EDL933 LEE as a query in Geneious Prime^®^. The sequences of the LEEs were extracted from corresponding genomes and aligned using Clustal Omega alignment in Geneious Prime^®^. A neighbor-joining consensus tree was constructed using the LEE in strain EDL933 as an outgroup with the following parameters: Genetic Distance Model, Jukes-Cantor; Resampling tree method: Bootstrap; Number of Replicates: 10,000; Support Threshold: 50%. The length of each LEE is indicated in parentheses. Subtypes of intimin gene *eae* were identified by BLASTn search of a database with 19 subtypes of *eae*. (**B**) Alignment of representative *E. albertii* LEEs. Representative *E. albertii* LEEs were aligned with the EDL933 LEE using Clustal Omega method. Green triangles represent the annotated CDSs, and red blocks represent LEE polycistronic operons. The identity of the consensus sequence is color-coded (green: 100% identity; brown: at least 30% and under 100% identity; and red: below 30% identity).

Regardless of the isolation source, all *E. albertii* strains examined carried an LEE, with a size ranging from 37,521 bp to 52,723 bp. Sequence alignments of LEEs revealed three clusters (Figure 2A). Cluster I contained LEEs from seven *stx*_2f_-positive wild bird strains and five clinical strains, among which three were *stx*_2f_-positive. All strains within this cluster carried an intimin sigma subtype gene except for the clinical strain 2012EL-1823, which encoded an intimin alpha subtype. Cluster II contained LEEs from three wild bird strains and the chicken carcass isolate 2014C-4356. The intimin subtypes encoded by the strains within this cluster included alpha, sigma, upsilon, and xi. Cluster III contained four clinical strains, including an *stx*_2f_-positive strain and encoded intimin subtypes of alpha, iota, omicron, and tau. Unlike the LEE in the EHEC strain EDL933, all LEEs in the *E. albertii* strains were inserted downstream of the tRNA gene *pheU*. However, all five polycistronic operons (LEE1-LEE5) defined in the EDL933 LEE were conserved in the LEEs of the *E. albertii* strains examined (Figure 2B). Variation in the LEE was mostly detected at the two junction regions, especially in the region between the LEE4 and the LEE integration site. The accessory genes located within these variable regions included mainly the mobile genetic element (MGE)-related genes, T3SS effector genes, transcriptional regulator genes, and heavy metal resistance genes.

**Figure 3:**
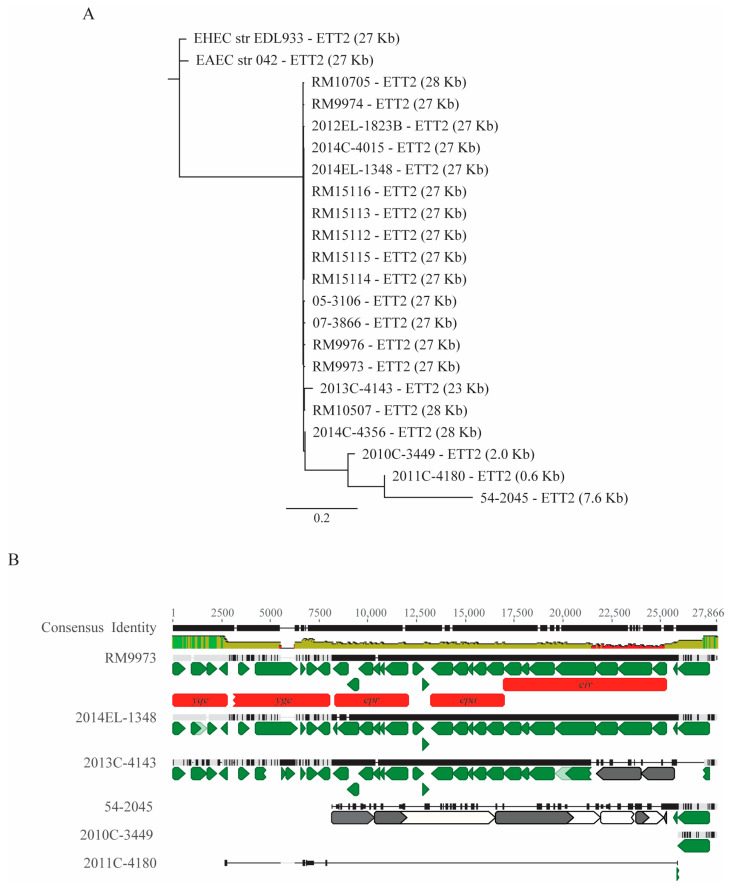
Sequence analyses of ETT2 in *E. albertii*. (**A**) Phylogeny of *E. albertii* ETT2s. The ETT2s in *E. albertii* strains were identified by BLASTn search of a database containing all genomes examined in this study using DNA sequence of EDL933 ETT2 as a query in Geneious Prime^®^. The sequences of the ETT2s were extracted from corresponding genomes and aligned using Clustal Omega alignment, and a neighbor-joining consensus tree was constructed using EDL933 ETT2 as an outgroup with the following parameters: Genetic Distance Model, Jukes-Cantor; Resampling tree method: Bootstrap; Number of Replicates: 10,000; Support Threshold: 50%. (**B**) Alignment of representative *E. albertii* ETT2s. Six representative *E. albertii* ETT2s were aligned using Clustal Omega method. Green triangles represent the annotated CDSs, and red blocks represent genes with annotated functions. Grey triangles represent hypothetical genes. The triangles with an overlapping smaller triangle represent genes carrying mutations that result in a premature stop codon. The identity of the consensus sequence is color-coded (green: 100% identity; brown: at least 30% and under 100% identity; and red: below 30% identity).

Besides LEE, some *E. coli* strains carry homologs of ETT2 (*E. coli* Type Three Secretion System 2), a 29 Kb PAI exhibiting sequence similarity with the *Salmonella* pathogenicity island 1 (SPI-1) [18]. Homologs of a complete ETT2 were identified in all ten *E. albertii* wild bird strains (Figure 3A). The majority of genes involved in biogenesis of T3SS apparatus were present on the ETT2s in the wild bird strains. A complete ETT2 was also detected in five clinical strains and the chicken isolate, 2014C-4356, while an incomplete ETT2 was present in the other strains (Figure 3A). In the clinical strain 2011C-4180, none of the ETT2-encoded genes were detected. There was only a 623 bp intergenic DNA fragment bordered by the tRNA gene *glyU* and an IS1 family transposase gene, while in clinical strain 2013C-4143, deletions in multiple ETT2 genes were observed, resulting in an incomplete ETT2 (~23 Kb) carrying various deletions in the *eiv* gene cluster (Figure 3B). Similar to strain EDL933, all ETT2s in the *E. albertii* were inserted downstream of tRNA gene *glyU*.

#### 3.2.2. OI-122 

**Figure 4:**
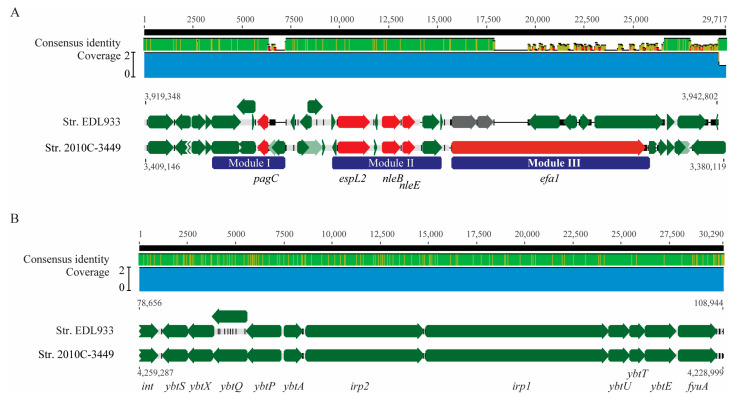
Sequence analyses of OI-122 and HPI in *E. albertii***.** (**A**) Pairwise comparison of OI-122 in strains EDL933 and 2010C-3449. The sequences were aligned using Clustal Omega in Geneious Prime^®^. Green arrows represent genes, while red arrows represent virulence genes located on three modules (purple blocks). (**B**) Pairwise comparison of HPI in strains EDL933 and 2010C-3449. The sequences were aligned using Clustal Omega in Geneious Prime^®^. The identity of the consensus sequence is color-coded (green: 100% identity; brown: at least 30% and under 100% identity; and red: below 30% identity).

The OI-122 in strain EDL933 is composed of three virulence gene modules: module I carries the virulence gene *pagC*, module II carries non-LEE effector genes *espL2*, *nleB*, and *nleE*, and module III carries a gene encoding the lymphostatin Efa1/LifA protein (Figure 4A). A complete OI-122 homolog was detected in clinical strain 2010C-3449 and inserted immediately downstream of tRNA gene *pheV*. All virulence genes located on the EDL933 OI-122 were present on the OI-122 of the *E. albertii* strain 2010C-3449. In other *E. albertii* strains, although no homologs of OI-122 were detected, some of the OI-122 virulence genes were detected, such as *nleB* and *nleE* in the clinical strains 2012EL-1823B and 2014C-4014 and in the wild bird strains RM9973, RM9974, RM10507, and RM10705; and *espL2*, *nleB*, and *nleE* in the clinical strains 07-3866, 2014C-4015, and 2014EL-1348 and in the five wild bird strains belonging to ST7971 (Strains RM15112-RM15116).

#### 3.2.3. HPI 

HPI, originally identified in *Yersinia* species [50], is involved in iron storage and uptake and has been detected in diverse STEC strains [51]. Like the OI-122, a complete homolog of HPI was only detected in the clinical strain 2010C-3449, which exhibited >99% sequence identity to the *Yersinia* HPI (Figure 4B). In the other *E. albertii* strains, homologs to any of the HPI genes were not detected.

#### 3.2.4. T6SS 

**Figure 5:**
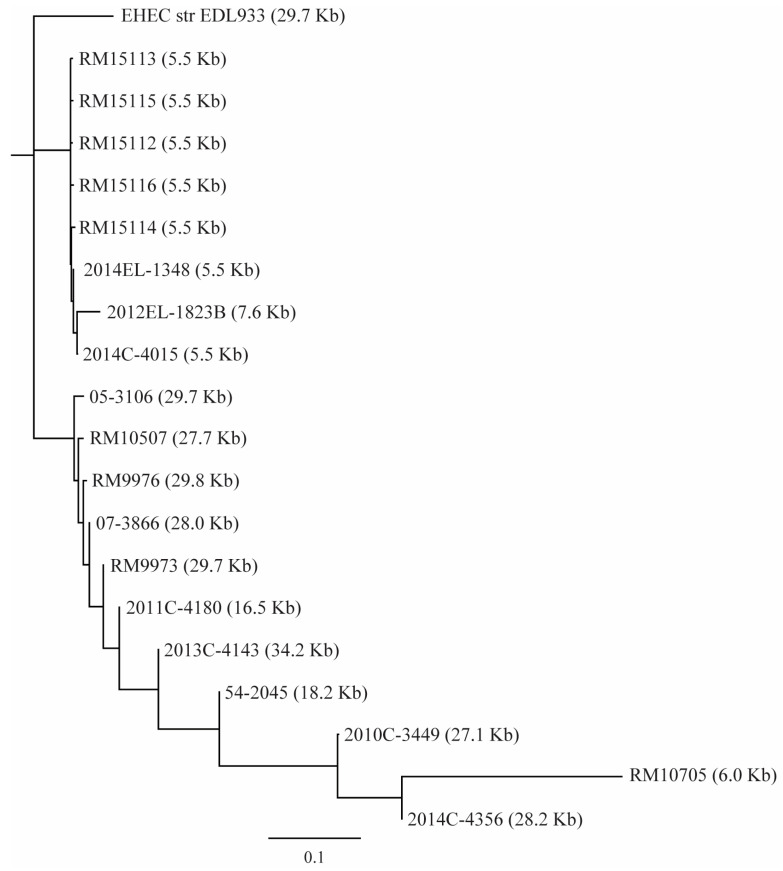
T6SSs in *E. albertii* strains. Phylogenetic analyses of T6SS-2. Sequences of *E. albertii* T6SS gene clusters and the T6SS gene cluster from EHEC strain EDL933 were aligned using Clustal Omega in Geneious Prime^®^ software Version 2023.2.1. A consensus tree was constructed using Geneious Tree Builder with the following settings: Genetic Distance Model: Jukes-Cantor; Tree Build Method: Neighbor-Joining; Outgroup: EDL933 T6SS; Resampling Method: Bootstrap; Random Seed: 326,544; Number of Replicates: 10,000. The length in parentheses represents the length of DNA fragment carrying T6SS genes. Strain RM9974 was not included in the analyses due to absence of T6SS gene cluster.

A BLASTn search of the three T6SS GIs, T6SS-1 from the EAEC strain 42 (~33 Kb), T6SS-2 from strain EDL933 (~30 Kb), and T6SS-3 from StxEAEC strain 2011C-3493 (~18 Kb) revealed homologs of T6SS-2 in the majority of *E. albertii* strains (Figure 5). A complete or a nearly complete T6SS-2 was detected in the clinical strains 05-3106, 07-3866, 2010C-3449, 2013C-4143, the chicken strain 2014C-4356, and the wild bird strains RM9973, RM9976, and RM10507. An incomplete T6SS-2 was detected in other *E. albertii* strains except in strain RM9974, where the corresponding chromosome site was occupied by an ISAs1 family transposases gene (locus tag, FYK18_08020). Sequence analysis of T6SS-2 revealed two large clades, one containing the five wild bird strains carrying ST7971 (strains RM15112-RM15116) and three *stx*_2f_-positive clinical strains 2012EL-1823B, 2014C-4015, and 2014EL-1345. The T6SS-2 GIs in this clade were all incomplete (5.5 or 7.6 Kb), only containing genes that encode the T6SS tip protein VgrG and the large type IV secretion protein Rhs. The T6SS-2 GIs in Clade II exhibited greater sequence diversity compared with the T6SS genes in Clade I, as demonstrated by the multiple branches in Clade II (Figure 5). A majority of the strains in this clade carried a complete T6SS gene cluster.

### 3.3. Detection of E. coli virulence Genes in E. albertii 

A total of 55 genes encoding T3SS effectors reported mainly in STEC and EPEC were retrieved from VFDB and used as queries to search a database containing all *E. albertii* strains examined in this study (Supplementary Table S1). The number of detected T3SS effector genes in *E. albertii* strains varied from 17 to 43 in clinical strains and from 28 to 39 in wild bird strains. The most common T3SS effector genes detected were *nleG* (nine alleles), *nleH* (two alleles), *nleF, map, espM* (two alleles)*, espO1* (two alleles)*, espH, espZ, espB, espG, and espC*. The genes *espM2*, *nleC*, *nleG-1*, *nleG-2*, *nleG5-1*, and *nleG6-1* appeared to be associated with the wild bird strains. 

A total of 30 *E. coli* genes encoding toxins or transporters involved in the toxin delivery were further examined in *E. albertii* strains (Supplementary Table S2). Among the eight genes encoding serine protease autotransporter toxins, homologs of *espC* and *espI* were detected in three clinical strains but not in any of the wild bird strains. In contrast, a gene encoding the vacuolating autotransporter toxin Vat was detected in all of the wild bird strains and in the majority of the clinical strains. The toxin B gene was also detected in all of the wild bird strains but only in seven of the clinical strains. The Shiga toxin genes, encoding the Stx2f subtype, were detected in all wild bird strains but only in four clinical strains. The cytolethal distending toxin gene *cdtABC* was detected in all *E. albertii* strains examined. Interestingly, multiple *cdtABC* genes were present in all *stx*_2f_-positive strains, while only a single copy was detected in all *stx*_2f_-negative strains. Genome mapping revealed that all wild bird strains and three clinical strains harbored two copies of *cdtABC*, and one of them was associated with a prophage (Supplementary Table S2). In the clinical strain 2012EL-1823B, there were three copies of *cdtABC* genes, and two of them were on the prophage genomes. Mutations were detected in the *cdtB* of the clinical strains 54-2045, 2011C-4180, and 2012EL-1823 and in the *cdtC* of the wild bird strain RM15114.

### 3.4. Stx2f-Converting Prophages and Production of Stx2f under Non-Inducing Condition 

**Figure 6:**
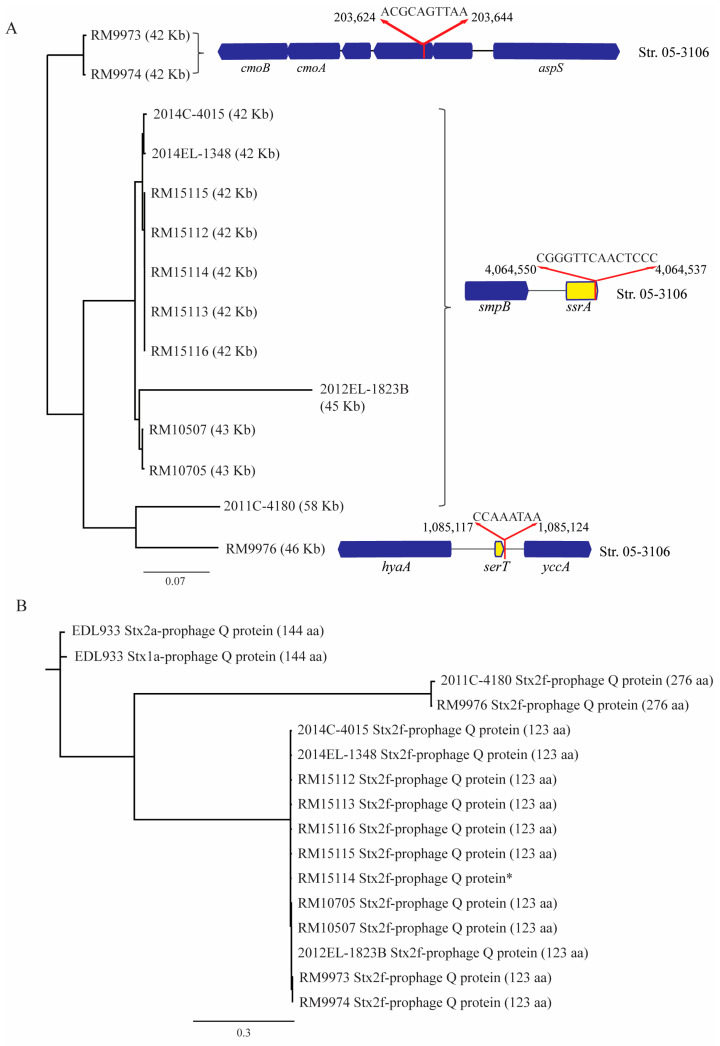
Sequence analyses of Stx2f-converting prophages in *E. albertii*. (**A**) Relatedness and chromosomal locations of Stx2f prophages. The genome size of each prophage is indicated in parentheses. The chromosomal insertion sites of Stx2f prophage are indicated with the corresponding positions in the clinical strain 05-3106. The putative integration sites are indicated by the red arrows. Yellow blocks refer to tmRNA and tRNA genes. Blue blocks refer to annotated genes. (**B**) Sequence analyses of antitermination protein Q genes located upstream of *stx*_2f_ coding sequences. The coding sequences of antitermination protein Q were aligned using Clustal Omega. A consensus tree was generated using neighbor-joining method with Jukes-Cantor for genetic distance model and rooted with the Q protein encoded by the Stx2a-prophage in EHEC strain EDL933. The Q protein size is indicated in parentheses. An asterisk indicates a truncated Q protein.

The genome size of Stx2f-prophages in *E. albertii* strains varied from 42 Kb to 58 Kb. Sequence alignment revealed three clusters, among which the largest cluster contained three clinical and seven wild bird strains (Figure 6A). The Stx2f-prophages in this group were all inserted within the transfer messenger RNA (tmRNA) gene *ssrA*. The Stx2f-prophages in the wild bird strains RM9973 and RM9974 were highly similar and were inserted within a hypothetical gene located upstream of the *aspS* gene (Figure 6A). The Stx2f-prophage in the wild bird strain RM9976 was inserted downstream of the tRNA gene *serT*. The Stx2f-prophage in the clinical strain 2011C-4180 displayed the highest sequence similarity with the Stx2f-prophage in strain RM9976 but was inserted within the *ssrA* gene, like the majority of *stx*_2f_-positive strains examined in this study. 

Examination of the DNA sequences upstream of *stx*_2f_ coding sequences in *E. albertii* did not identify the putative late promoter *P*_R’_ or the antitermination Q protein binding site *qut.* The majority of STEA strains carried a 123 aa antitermination Q protein, which exhibited about 50% identity with the Q protein (144 aa) encoded by the Stx2a-prophage in strain EDL933 (Figure 6B). A larger antitermination Q protein (276 aa) was revealed in the clinical strain 2011C-4180 and in the wild bird strain RM9976, exhibiting only about 11% identity with the Q protein in strain EDL933. 

Spontaneous production of Stx2f in *E. albertii* was further evaluated, and the results showed a large variation in Stx2f production among the STEA strains grown in rich growth medium to the stationary phase. The greatest production was observed in wild bird strain RM9974 (28.5 ng/mL), followed by wild bird strain RM9973 (5.6 ng/mL), and the clinical strain 2011C-4180 (3.3 ng/mL). Spontaneous production of Stx2f was in the range of 0.1–1.0 ng/μL in the majority of *E. albertii* strains and undetectable in the clinical strain 2012EL-1823B (Table 2). Similarly, production of Stx2f in the three STEC strains under non-inducing conditions varied greatly. In strain RM7007 from pigeon and strain RM10064 from water, the Stx2f was below 5.0 ng/mL, while in another isolate from water, RM16643, Stx2f production was not statistically different from the *stx*-negative strains (Table 2). 

### 3.5. Cytotoxicity in E. albertii Strains 

The Stx-mediated cytotoxicity of the STEA strains was assessed by using the mammalian host Vero-d2EGFP cells and bacterial cell-free culture supernatants. In this Vero cell-based assay, incubation with a high concentration of active Stx resulted in low levels of fluorescence in the Vero-d2EGFP cells, and the detected fluorescence levels in the examined wild bird STEA strains were compared to those of the *stx*-negative *E. coli* strains, *stx*_2f_-positive *E. coli* strains, and STEC outbreak strains associated with HUS in infected humans (Table 2). For the ten wild bird STEA strains, the Vero-d2EGFP-based cytotoxicity was all significantly higher than the *stx*-negative *E. coli* strains (One-way ANOVA, *p* = 0.0005); compared with the *stx*_2f_-positive *E. coli* strains, strains RM9973, RM9974, RM10705, RM15112, RM15113, and RM15115 exhibited significantly higher cytotoxicity (One-way ANOVA, *p* < 0.05). Although One-Way ANOVA revealed no significant difference in the average of fluorescence values between the wild bird STEA strains and the three STEC outbreak strains (EDL933, RM6103, and RM12238), pairwise strain comparison suggested that strains RM9976, RM10507, and RM15116 displayed lower cytotoxicity than the three outbreak strains (Dunnett’s test, *p* < 0.05). Although the average fluorescence values detected for the wild bird strains RM9974 and RM10705 were lower than for any of the three STEC outbreak strains (EDL933, RM6013, and RM12238), the differences were not statistically significant (Dunnet’s test, *p* = 0.0605). When examining the clinical STEA, strains 2011C-4180, 2014C-4015, and 2014EL-1348 exhibited higher cytotoxicity than those of *stx*_2f_-negative *E. albertii* strains (paired *t*-test, *p* < 0.05). There was no significant difference in the average of fluorescence values between the strain 2012-EL-1823B (*stx*_2f_-positive) and any of the *stx*_2f_-negative *E. albertii* strains (Table 2).

## 4. Discussion

*E. albertii* was recognized almost 20 years ago to be closely related to strains of *Shigella boydii* and estimated to have separated from an *E. coli*-like ancestor about 28 million years ago [3]. Recent genomic-based studies revealed remarkable genetic diversity within this species [9,12,17,25], providing supportive evidence for the evolutionary radiation of this species in exploiting a wide range of ecological niches. Consistently, our comparative genomic analyses of 20 complete *E. albertii* genomes revealed a large pangenome and a very small core genome, supporting that, like *E. coli*, *E. albertii* holds an open pangenome and evolves more rapidly compared to the species with closed pangenomes [52]. Among the ten wild bird strains, four Oregon junco strains and one white-breasted nuthatch strain carry the same sequence type (ST7971) and are phylogenetically closely related to clinical strains 2014C-4015 (ST5992), 2014EL-1348 (ST5990), and 2012EL-1823 (ST5983) (Supplementary Figure S1), consistent with the core gene-based phylogeny (Figure 1). Therefore, these strains are within the Clade II lineage, according to a previous report on the phylogenetic diversity and population structure of *E. albertii* [53]. The other wild bird strains that are phylogenetically highly related to the above clinical strains include one American crow strain (RM9974, ST2682) and one brown-headed cowbird strain (RM10705, ST9018) since both core genome- and MLST-based phylogenetic analyses placed both strains in the same cluster as the above three clinical strains (Figure 1 and Supplementary Figure S1).

A search of EnteroBase revealed that strains belonging to ST7971 have been isolated previously from spinach (BioSample number: SAMN12230424) and stool samples (BioSample: SAMN08199346), and both were reported as *E. coli* isolates. ST5969, ST5983, ST5992, and ST7833 differ from ST7971 with one locus. Strains belonging to one of the above four STs include strains recovered from kale leaves (BioSample: SAMN04422427), human blood (BioSample: SAMN10875686), and a diarrheal patient in the United Kingdom (Strain 367208). A search of EnteroBase revealed 12 strains belonging to ST2682, same as the American crow strain RM9974, among which a majority of these were from wild birds isolated in Japan, Australia, and the United Kingdom. Of the two U.S. strains carrying ST2682, one was isolated from vegetable (BioSample: SAMN04429912) while the other one was a clinical isolate (BioSample: SAMN32772508). Strains belonging to ST9018, same as the brown-headed cowbird strain RM10705, included a river water isolate (BioSample: SAMN08797203) and a municipal water isolate (BioSample: SAMN08797189) reported by Agriculture and Agri-Food Canada and a clinical strain (BioSample: SAMN12100513) reported by the CDC. The sequence type classified for the American crow strain RM9973, ST6057, was also classified for a clinical *E. albertii* isolate reported in 2016 (BioSample: SAMD00006218). The presence of the clinically relevant *E albertii* genotypes recovered from various environmental samples may suggest diverse sources of potential clinical infections.

Current knowledge about the prevalence, epidemiology, and pathogenesis of *E. albertii* is limited due to historical misclassification of this species as *E. coli*, *Shigella boydii*, or *H. alvei* [7,8]. The best-known virulence determinants in *E. albertii* are located on the LEE, similar to the LEEs in EPEC/EHEC, a PAI containing genes (*eae* and *tir*) responsible for the initial adherence of pathogen cells to the host epithelial cell surfaces as well as the genes encoding T3SS apparatus proteins and effectors. Like EPEC/EHEC strains, *E. albertii* appears to have acquired an LEE via horizontal gene transfer since the GC contents of the LEEs (38.6–40.7%) examined in this study were all much lower than the average GC contents of the corresponding chromosomes (49.5–50.1%). All LEEs in the *E. albertii* strains examined were uniformly integrated next to the tRNA gene *pheU*, and this observation was consistent with a previous report stating that the *pheU* locus is a preferable integration site for LEE in *E. albertii* [7]. Furthermore, diverse intimin subtypes were detected. The dominant subtype was sigma, present in seven wild bird strains and five clinical strains. Other subtypes included alpha, upsilon, and xi in the wild bird strains and alpha, iota, nu, omicron, and tau in the clinical strains. As previously documented, intimin-mediated pathogen adherence is the first step in the formation of A/E lesions during the pathogenesis of attaching-and-effacing *E. coli* (AEEC) strains [54]. Additionally, intimin is thought to be responsible for host specificity and tissue cell tropism because EHEC strains harboring different intimin subtypes exhibited different colonization patterns [55]. It is unknown if intimin subtype sigma confers *E. albertii* with any host colonization specificity, such as birds. Currently, there are nearly 40 intimin subtypes reported, and more studies are needed to understand the prevalence, host/tissue specificity, and clinical relevance of other intimin subtypes.

The other well-known virulence factor in *E. albertii* is CDT, a tripartite holotoxin that induces eukaryotic cell cycle arrest and eventually leads to apoptosis [56]. Production of CDT is thought to be associated with increased persistence, invasion, and disease severity in various bacterial pathogens [57,58]. Because multiple copies of *cdt* genes were detected in all *stx*_2f_-positive *E. albertii* strains, we further examined the chromosome locations and the relatedness of the *cdt* operons in the examined bacterial strains. Interestingly, in all except the wild bird strain RM9976, one set of *cdtABC* was located on the Stx2f-prophage genome. In strain RM9976, the *cdtABC* genes were on a prophage (chromosome location 1,103,419-1,169,082) but not the Stx2f-prophage. In *E. coli*, CDT has been classified into five subtypes based on the sequence variations in *cdtB*, although a published study suggested that *cdtB*-II was associated with *E. albertii* [56,59]. Sequence comparison of the *E. albertii cdtB* genes with the *E. coli cdtB* I-V subtypes revealed that all prophage-encoded *cdtB* genes were grouped with the *cdtB*-I while others were grouped with *cdtB*-II or *cdtB*-III/V (clinical strains 2013C-4143, 05-3106, and 2011C-4180) (Supplementary Figure S2), supporting a previous report that CDT type I and IV genes are framed with lambdoid prophage genes [56].

Consistent with previous reports documenting Stx2f to be the predominant subtype in *E. albertii* [7,11,33,60], all wild bird *E. albertii* strains examined in this study carried phage-borne *stx*_2f_ genes. The complete genome sequences from this study allowed the Stx2f-prophage integration sites to be mapped and compared with the ones in *E. coli*. The most common site, the tmRNA gene *ssrA*, is also commonly utilized in *E. coli* and in other members of the family *Enterobacteriaceae* for integration of bacteriophages, including Stx-phages and phages carrying other virulence genes, e.g., T3SS effector genes [61,62,63]. Although the tRNA gene *serT* has not been reported as an Stx-phage integration site in *E. coli*, the tRNA gene *serU* is known for Stx-phage integration in an *E. coli* O121 strain [43]. The insertion sites in the wild bird strains RM9973 and RM9974 are located within a hypothetical gene that is widespread in *E. albertii* and *E. coli* populations. 

The substantial variation in Stx2f production observed in this study could not be explained by the difference in regulatory elements of the *stx*_2f_ genes or the genetic makeup of Stx2f-prophages. The Stx2f-prophages in strains RM9973 and RM9974 were nearly identical and utilized the same chromosome integration site. Furthermore, the DNA sequences upstream of the *stx*_2f_ coding sequence, as well as the gene encoding antitermination protein, were identical in the two strains. The vast difference in the spontaneous Stx2f production between the two strains may be due to the difference in the physiological state of host bacteria, which is a known factor that influences Stx production [64,65]. Strain variation in spontaneous induction of Stx1a and Stx2a was reported in a group of STEC O145:H28 strains carrying the Stx-prophages within the same lineage [66]. Two groups of antitermination protein were identified in *E. albertii* strains examined in this study, and the majority of STEA strains carried a 123 aa protein belonging to pfam 06350, while strains RM9976 and 2011C-4180 carried a 276 aa protein belonging to pham 03589. Further studies are needed to understand whether, like many Stx-prophages in *E. coli*, Stx2f-prophages in *E. albertii* are agent inducible, the conditions that induce the production of Stx2f, as well as the role of the antitermination proteins in the production of Stx2f. 

Given that *stx*_2f_-positive STEC strains have rarely been associated with HUS, the clinical significance of STEA is likely underestimated due to the misclassification of this pathogen as *E. coli*. In the first report of a clinical *E. albertii* strain expressing Stx2f, a re-examination of previously identified EPEC or EHEC strains using routine classification systems adopted in clinical labs revealed that 15% of them were *E. albertii* [7]. Stx2f is thought to be less toxic to mammalian cells than Stx2a but more stable at low pH and high temperature, according to a study using purified toxins [67]. Our study uncovered that the majority of STEA wild bird strains exhibited higher cytotoxicity than the *stx*_2f_-positive *E. coli* strains, and several of them displayed a comparable cytotoxicity level with the *stx*_2a_-positive EHEC outbreak strains and an EHEC strain carrying both *stx*_1a_ and *stx*_2a_, consistent with the finding that the Stx2f-expressing *E. albertii* strain AKT5 had a similar level of cytotoxicity as the EHEC outbreak strain Sakai [68]. The increased cytotoxicity in the *stx*_2f_-positive *E. albertii* strains might be attributed to other toxins, such as CDT. CDT produced from *E. coli* and *Campylobacter* spp. can induce cellular distension, cell cycle arrest, and cell death in the mammalian Vero cells, although the receptors mediating the entry of CDT are unknown [58]. A BLASTn search of the genomes of the EHEC strains examined in this study and the genome of the strain Sakai did not identify any homologs of *cdtABC* genes. Further genetic and biochemical studies are required to reveal if CDTs have contributed to the increased cytotoxicity in the STEA strains detected in our study. 

Rapid identification of foodborne pathogens that have the potential to cause severe human disease is one of the biggest challenges in food safety. For STEC, a combination of Stx subtypes and the adherence capacity has been used traditionally to predict the pathogenicity potential of a strain [69]. Because the subtypes Stx1a, Stx2a, and Stx2d are most frequently implicated in causing severe human illness, including HUS [70], strains that possess one of the above Stx subtypes and also express the intimin adhesin, such as in EHEC strains, are considered high-risk STEC strains. Although the majority of the STEA strains characterized to date have been found to carry *stx*_2f_ genes only, an *stx*_2a_-positive *E. albertii* strain was isolated from a patient who had developed bloody diarrhea [22], suggesting that, similarly to STEC, *E. albertii* acquires virulence genes via horizontal gene transfer in a lineage-independent manner. In fact, our study revealed the significant contribution of MGEs in shaping the virulence gene repertoire in *E. albertii*. Besides LEE, pathogenicity islands OI-122 and HPI were detected. In addition to genes encoding Stx and CDTs, genes encoding the T3SS effectors were frequently associated with the prophage genomes in *E. albertii*. Although the enterohemolysin genes *(hlyCABD)*, often present on the large virulence plasmid in EHEC strains, were not detected in *E. albertii* strains examined in this study, *hlyCABD*-positive *E albertii* strains were reported in China [25]. In addition to vast genetic diversity, phenotypic variation, including Stx2f production and cytotoxicity revealed in our study, implies that *E. albertii* strains are likely varying in their pathogenicity, even among the strains belonging to the same genomic lineage. Loss-of-function mutations in genes encoding master transcriptional regulators are known to contribute to the strain variation in stress resistance and biofilm formation in EHEC strains. Knowledge about the regulation of virulence gene expression in *E. albertii* is limited. Further studies are needed to illustrate the impact of bacterial and environmental factors on the expression of virulence genes and pathogenicity traits, as well as to determine how ecological niches may impose a selective pressure for these traits. Such information will be valuable in developing a better understanding of the pathogenesis of *E. albertii* and an effective mitigation strategy for the detection, identification, and control of this emerging avian and human pathogen. 

## Figures and Tables

**Table 1:** Genomic characteristics of *E. coli* and *E. albertii* strains used in this study.

Strains ^1^	Sources/ Year of Isolation	Chromosome (bp)/ GenBank Accession #	Plasmids (bp)/ GenBank Accession #	Sequence Type ^2^ (ST)	*stx* Genes	References
*E. coli*						
EDL933	Ground beef/1982	5,528,445/ AE005174.2	92,077/AF074613.1	11	*stx*_1a_ + *stx*_2a_	[29,30]
MG1655	Human/1922	4,641,652/ U00096.3	None	10	None	[31]
*E. albertii*						
RM9973	American crow (*Corvus brachyrhynchos*)/2009	4,648,335/ CP043271.1	p1, 105,082/CP043273.1; p2, 102,351/CP043272.1	ST6057 *	*stx*_2f_	This study
RM9974	American crow (*Corvus brachyrhynchos*)/2009	4,863,808/ CP043266.1	p1, 132,007/CP043270.1; p2, 99,091/CP043269.1; p3, 96,650/CP043268.1; p4, 27,507/CP043267.1	ST2682	*stx*_2f_	This study
RM9976	American crow (*Corvus brachyrhynchos*)/2009	4,976,140/ CP043262.1	p1, 200,52/CP043265.1; p2, 85,807/CP043264.1; p3, 84,014/CP043263.1	ST8692	*stx*_2f_	This study
RM10507	Brown-headed cowbird (*Molothrus ater*)/2009	5,007,500/ CP043258.1	p1, 102,034/CP043261.1; p2, 99,002/CP043260.1; p3, 42,515/CP043259.1	ST10004 *	*stx*_2f_	This study
RM10705	Brown-headed cowbird (*Molothrus ater*)/2009	4,904,046/ CP045690.1	p1, 117,701/CP045688.1; p2, 102,214/CP045689.1	ST9018	*stx*_2f_	This study
RM15112	Oregon Junco (*Junco hyemalis*)/2011	4,712,580/ CP043254.1	p1, 136,729/CP043257.1; p2, 97,372/CP043256.1; p3, 62,266/CP043255.1	ST7971	*stx*_2f_	This study
RM15113	Oregon Junco (*Junco hyemalis*)/2011	4,712,547/ CP043250.1	p1, 136,729/CP043253.1; p2, 97,374/CP043252.1; p3, 62,269/CP043251.1	ST7971	*stx*_2f_	This study
RM15114	Oregon Junco (*Junco hyemalis*)/2011	4,712,362/ CP043246.1	p1, 136,747/CP043249.1; p2, 97,369/CP043248.1; p3, 62,259/CP043247.1	ST7971	*stx*_2f_	This study
RM15115	White-Breasted Nuthatch (*Sitta carolinensis*)/2011	4,712,540/ CP043242.1	p1, 136,727/CP043245.1; p2, 97,365/CP043244.1; p3, 62,256/CP043243.1	ST7971	*stx*_2f_	This study
RM15116	Oregon Junco (*Junco hyemalis*)/2011	4,712,500/ CP043238.1	p1, 136,731/CP043241.1; p2, 97,363/CP043240.1; p3, 62,266/CP043239.1	ST7971	*stx*_2f_	This study
2014C-4356	Chicken Carcass/2009	4,852,165/ CP024282.1	p1, 40,461/CP024283.1; p2, 59,626/CP024284.1; p3, 127,606/CP024285.1; p4, 113,727/CP024286.1; p5, 124,142/CP024287.1; p6, 19,118/CP024288.1	ST7415	None	[32]
05-3106	Human/2005	4,719,735/ CP030778.2	P1, 56,603/CP030779.2; P2, 80,632/CP030780.2	ST4619	None	[33]
07-3866	Human/2007	4,940,006/ CP030781.1	104,269/CP030782.1	ST7414	None	[33]
54-2045 (NCTC 9362)	Human/1954	4,551,125/ CP034213.1	40,180/CP034214.1	ST413	None	[33]
2010C-3449	Human/2010	4,923,641/ CP034212.1	None	ST5390	None	[33]
2011C-4180	Human/2011	4,790,629/ CP126912	p1, 48,807/CP126913 p2, 98,642/CP126914 p3, 151,845/CP126915	ST2681	*stx*_2f_	This study
2012EL-1823B	Human/2012	4,809,821/ CP030783.2	p1, 100,347/CP030784.2; p2, 81,130/CP030785.2; p3, 105,846/CP030786.2	ST5983	*stx*_2f_	[33]
2013C-4143	Human/2013	4,659,709/ CP030787.2	None	ST7960	None	[33]
2014C-4015	Human/2014	4,623,903/ CP034166.1	p1, 63,809/CP034165.1; p2, 96,264/CP034164.1; p3, 136,645/CP034167.1	ST5992	*stx*_2f_	[33]
2014EL-1348	Human/2014	4,662,779/ CP126908	p1, 62,344/CP126909 p2, 96,264/CP126910 p3, 136,695/CP126911	ST5990	*stx*_2f_	This study

^1^ The source and isolation year for strain MG1655 is based on the information available for its parental strain K-12 as described previously [34]. ^2^ The sequence type (ST) was determined using MLST 2.0 service at the Center for Genomic Epidemiology with the *Escherichia coli* # 1 configuration [35]. * The closest ST.

**Table 2:** Production of Stx2f and cytotoxicity in *E. albertii*.

Strains (Serotype) ^1^	Sources	*stx* Subtype	Stx2f (ng/mL)	Cytotoxicity ^2^
*E. albertii*				
RM9973	American crow (*Corvus brachyrhynchos*)	*stx*_2f_	5.6 ± 0.3	++
RM9974	American crow (*Corvus brachyrhynchos*)	*stx*_2f_	28.5 ± 1.6	++++
RM9976	American crow (*Corvus brachyrhynchos*)	*stx*_2f_	0.8 ± 0.2	+
RM10507	Brown-headed cowbird (*Molothrus ater*)	*stx*_2f_	0.1 ± 0.0	+
RM10705	Brown-headed cowbird (*Molothrus ater*)	*stx*_2f_	1.1 ± 0.1	+++
RM15112	Oregon Junco (*Junco hyemalis*)	*stx*_2f_	0.6 ± 0.0	++
RM15113	Oregon Junco (*Junco hyemalis*)	*stx*_2f_	0.3 ± 0.3	+++
RM15114	Oregon Junco (*Junco hyemalis*)	*stx*_2f_	0.4 ± 0.1	++
RM15115	Oregon Junco (*Junco hyemalis*)	*stx*_2f_	0.2 ± 0.0	++
RM15116	White-Breasted Nuthatch (*Sitta carolinensis*)	*stx*_2f_	0.6 ± 0.2	++
2014C-4356	Chicken Carcass	*stx* negative	0.1 ± 0.1	-
2011C-4180	Human	*stx*_2f_	3.3 ± 0.4	++
2012EL-1823B	Human	*stx*_2f_	0.0 ± 0.0	-
2014EL-1348	Human	*stx*_2f_	0.3 ± 0.1	+
2014C-4015	Human	*stx*_2f_	0.8 ± 0.6	+
05-3106	Human	*stx* negative	0.0 ± 0.0	-
07-3866	Human	*stx* negative	0.0 ± 0.0	-
54-2045	Human	*stx* negative	0.0 ± 0.0	-
2010C-3449	Human	*stx* negative	0.1 ± 0.0	-
2013C-4243	Human	*stx* negative	0.0 ± 0.0	-
*E. coli*				
RM7007	Pigeon (*Columba livia*)	*stx*_2f_	3.9 ± 0.1	+
RM10064	Water	*stx*_2f_	1.2 ± 0.1	++
RM16643	Water	*stx*_2f_	0.0 ± 0.0	-
RM5034 (K-12 strain ATCC#29425)	Human	*stx* negative	0.0 ± 0.0	-
RM4876 (O157:H7)	Water	*stx* negative	NA	-
RM1273 (O157:H7)	Human	*stx* negative	NA	-
EDL933 (O157:H7)	Ground beef	*stx*_1a_ + *stx*_2a_	NA	+++
RM6013 (O157:H7)	Human	*stx*_2a_	NA	+++
RM12238 (O145:H28)	Human	*stx*_2a_	NA	+++

^1^ Strain RM6013 is linked to the 2006 spinach-associated outbreak in the U.S.; strain RM12238 is linked to a 2010 romaine lettuce-associated outbreak in the U.S. ^2^ The cytotoxicity of each strain is classified by the relative EGFP fluorescence at the time of reading. ++++, 0–20%; +++, 20–40%; ++, 40–60%; +, 60–80%; -, >80%. The %EGFP and the cytotoxicity are inversely proportional.

## Data Availability

The data presented in this study are openly available in GenBank under the BioProject numbers PRJNA561565 and PRJNA218110. The GenBank accession numbers are listed in Table 1.

## Supplementary Materials

The following supporting information can be downloaded at: https://www.mdpi.com/article/10.3390/microorganisms11112803/s1, Table S1. Detection of *E. coli* T3SS effector genes in *E. albertii*. Table S2. Detection of toxin-related genes in *E. albertii*. Figure S1. Phylogeny of *E. albertii* strains based on sequences of the MLST loci. The concatenated sequences of the seven loci (*adk*, *fumC*, *gyrB*, *icd*, *mdh*, *purA*, and *recA*) were aligned using Clustal Omega, and the consensus sequence was used to construct phylogeny tree in Geneious Prime^®^ as detailed in Materials and Methods. The sequence type (ST) was determined using MLST 2.0 service at the Center for Genomic Epidemiology with the *Escherichia coli* #1 configuration. Figure S2. Phylogeny of *E. albertii cdtB*. Boldface indicates reference genes for five subtypes. The GenBank accession numbers are shown in parentheses. The *cdtB* gene located on the Stx2f-prophage in strain 2012EL-1823 was not included in the analyses due to a large insertional mutation in the *cdtB* gene (Loucs tag, AYN53_RS14910).
