# Supplementary material for: Genomic and Phenotypic Characterization of Shiga Toxin-Producing Escherichia albertii Strains Isolated from Wild Birds in a Major Agricultural Region in California
Source: Microorganisms. 2023 Nov 18;11(11):2803. doi: 10.3390/microorganisms11112803 (PMC10673567; doi:10.3390/microorganisms11112803)
Supplement: Supplementary file 1 [file microorganisms-11-02803-s001.zip › microorganisms-2641655-supplementary.pdf]

Table S1. Detection of *E. coli* T3SS effector genes in *E. albertii*.

Gene	Strain/pathotype	#Locus tag	Clinical Strains									Chicken strain	Wild bird strains									
			05-3106	07-3866	54-2045	2010C-3449	2011C-4180	2012EL-1823B	2013C-4143	2014C-4015	2014EL-1348	2014C-4356	RM9973	RM9974	RM9976	RM10507	RM10705	RM15112	RM15113	RM15114	RM15115	RM15116
cif	*E. coli* O127:H6 str. E2348/69 (EPEC)	E2348C_RS03860	0	0	0	0	1	1	0	0	0	0	0	0	0	0	0	0	0	0	0	0
espB	*E. coli* O157:H7 str. EDL933 (EHEC)	Z5105	1	1	1*	1	1	1	1	1	1	1	1	1	1	1	1	1	1	1	1	1
espC	*E. coli* O127:H6 str. E2348/69 (EPEC)	E2348C_RS15645	1*	1	1	1	1	1	1*	1	1	1	1	1	1	1	1	1	1	1	1	1
espF	*E. coli* O157:H7 str. EDL933 (EHEC)	Z5100	1	1	1	1	1	1*	1	1*	1*	1	1	1	1	1	1	1*	1*	1*	1*	1*
espG	*E. coli* O157:H7 str. EDL933 (EHEC)	Z5142	1	1	1	1	1	1	1	1*	1	1	1	1	1	1	1	1	1	1	1	1
espH	*E. coli* O157:H7 str. EDL933 (EHEC)	Z5115	1	1	1	1	1	1	1	1	1	1	1	1	1	1	1	1	1	1	1	1
espJ	*E. coli* O157:H7 str. EDL933 (EHEC)	Z3071	1	1	1	1	2+1*	1	1	1	1	1	1	1*	1	1*	1*	1	1	1	1*	1
espK	*E. coli* O157:H7 str. EDL933 (EHEC)	Z1829	1*	1*	2	2	0	1+1*	1	1	1	1*	1	1	1	1*	1	1	1	1	1	1
espL1	*E. coli* O157:H7 str. EDL933 (EHEC)	Z2749	0	0	0	0	0	0	0	0	0	0	0	0	0	0	0	0	0	1*	0	0
espL2	*E. coli* O157:H7 str. EDL933 (EHEC)	Z4326	0	1*	0	1	0	0	0	1	1	0	0	0	0	0	0	1	1	1*	1*	1*
espL4	*E. coli* O157:H7 str. EDL933 (EHEC)	Z5608	0	0	0	0	0	0	0	0	0	0	0	0	0	0	0	0	0	0	0	0
espM1	*E. coli* O157:H7 str. EDL933 (EHEC)	Z2565	0	0	0	0	0	0	0	0	0	0	1	0	0	0	0	0	0	0	0	0
espM2	*E. coli* O157:H7 str. EDL933 (EHEC)	Z3918	1*	1	1	1*	2	3	1*	2	2	1	0	1	2	3	3	2	2	2	2	2
espN	*E. coli* O157:H7 str. Sakai (EHEC)	ECs_1561	0	0	0	1	0	0	0	0	0	0	0	0	0	0	0	0	0	0	0	0
espO1-1	*E. coli* O157:H7 str. Sakai (EHEC)	ECs_1567	1	0	1	1	0	1	2	1	2	0	1	1	1	1	0	2	2	2	2	2
espO1-2	*E. coli* O157:H7 str. Sakai (EHEC)	ECs_1821	0	1	0	1	0	1	0	1	1	0	1	1	1	1	1	0	1	1	1	1
espR1	*E. coli* O157:H7 str. Sakai (EHEC)	ECs_2073	0	0	0	0	0	0	0	0	0	0	0	0	0	0	0	0	0	0	0	0
espR3	*E. coli* O157:H7 str. EDL933 (EHEC)	Z3023	0	0	0	0	0	0	0	0	0	0	0	0	0	0	0	0	0	0	0	0
espR4	*E. coli* O157:H7 str. EDL933 (EHEC)	Z3026	0	0	0	0	0	0	0	0	0	0	0	0	0	0	0	0	0	0	0	0
espW	*E. coli* O157:H7 str. EDL933 (EHEC)	Z3920	1*	1	1	1	0	1	0	1	1	1	1*	1	1	1	0	1	1	1	1	1
espX1	*E. coli* O157:H7 str. EDL933 (EHEC)	Z0025	0	0	1*	0	1*	1*	1*	1*	1*	0	0	1	0	0	1*	1*	1	0	1*	1*
espX2	*E. coli* O157:H7 str. EDL933 (EHEC)	Z1019	0	0	0	0	0	0	0	0	0	0	0	0	0	0	0	0	0	0	0	0
espX4	*E. coli* O157:H7 str. EC4115 (EHEC)	ECH74115_RS27410	0	0	0	0	0	0	0	0	0	0	0	0	0	0	0	0	0	0	0	0
espX5	*E. coli* O157:H7 str. EDL933 (EHEC)	Z5665	0	0	0	0	0	0	0	0	0	0	0	0	0	0	0	0	0	0	0	0
espX6	*E. coli* O157:H7 str. EDL933 (EHEC)	Z5935	0	1	1	1	1	1	1	1	1	1	1	1	1	1	1	1	1	1	1	1
espX7/nleL	*E. coli* O157:H7 str. EDL933 (EHEC)	Z1822	0	1	0	1	0	1	0	1	1	1	0	0	1	1	1	1	1	1	1	1
espY1	*E. coli* O157:H7 str. EDL933 (EHEC)	Z0065	0	0	0	0	0	0	0	0	0	0	0	0	0	0	0	0	0	0	0	0
espY2	*E. coli* O157:H7 str. EDL933 (EHEC)	Z0078	0	0	0	0	0	0	0	0	0	0	0	0	0	0	0	0	0	0	0	0
espY3	*E. coli* O157:H7 str. EDL933 (EHEC)	Z0521	1	1	0	1	1	1	1	1	0	1	1	1	1	1	1	1	1	1	1	1
espY4	*E. coli* O157:H7 str. EDL933 (EHEC)	Z5211	0	0	0	0	0	1	0	0	0	0	0	1	0	0	1*	1*	1*	0	0	0
espY5	*E. coli* O157:H7 str. EDL933 (EHEC)	Z5214	0	0	0	0	0	1	0	1	3*	0	0	0	0	0	1*	1*	1	1*	1*	1
espZ	*E. coli* O157:H7 str. EDL933 (EHEC)	Z5122	1	1	1	1	1	1	1	1	1	1	1	1	1	1	1	1	1	1	1	1
map	*E. coli* O157:H7 str. EDL933 (EHEC)	Z5113	1	1	1	1	1	1	1	1	1	1	1	1	1	1	1	1	1	1	1	1
nleA	*E. coli* O157:H7 str. EDL933 (EHEC)	Z6024	1*	1	0	1	1*	1	0	1	1	1	1	1	1	1	1	1*	1	1	1	1
nleB1	*E. coli* O157:H7 str. EDL933 (EHEC)	Z4328	0	1	0	2	0	1	0	1	1	1	1	1	0	1*	1	1	1	1	1	1*
nleB2	*E. coli* O157:H7 str. EDL933 (EHEC)	Z0985	0	0	1	0	1	0	0	0	0	0	0	0	0	0	0	0	0	0	0	0
nleB2-2	*E. coli* O55:H7 str. CB9615 (EPEC)	G2583_RS01765	1	0	0	0	0	0	1	0	0	0	0	0	0	0	0	0	0	0	0	0

nleC	*E. coli* O157:H7 str. EDL933 (EHEC)	Z0986	0	1	0	0	1*	1	0	1	1	1+1*	0	1	1	1	1	1	1	1	1	1
nleD	*E. coli* O157:H7 str. EDL933 (EHEC)	Z0990	0	0	0	0	1	1	0	1	1	0	0	0	1	0	0	1*	1*	1*	1*	1*
nleE	*E. coli* O157:H7 str. EDL933 (EHEC)	Z4329	0	1	0	1	0	1	0	1	1	1	1	1	0	1	1	1	1*	1	1	1
nleE-2	*E. coli* O127:H6 str. E2348/69 (EPEC)	E2348C_ RS05730	0	0	0	1	0	0	0	0	0	0	0	0	0	0	0	0	0	0	0	0
nleF	*E. coli* O157:H7 str. EDL933 (EHEC)	Z6020	1	1	1	1	1	1	1	1	1	1	1	1	1	1	1	1	1	1	1	1
nleG2-2	*E. coli* O157:H7 str. EDL933 (EHEC)	Z2339	0	1	0	1	2*	1	0	1	1	0	1	2	1	2	1	1	1	1	1	1
nleG2-4	*E. coli* O55:H7 str. CB9615 (EPEC)	G2583_ RS09785	1	0	0	1	0	0	0	0	0	1	0	0	0	0	0	0	0	0	0	0
nleG5-1	*E. coli* O157:H7 str. EDL933 (EHEC)	Z2337	1*	3	1	1+1*	0	3+1*	1	2	2+1*	3	2	4+1*	3+1*	4	1+1*	2+1*	2+1*	2+1*	2+1*	2+1*
nleG6-1	*E. coli* O157:H7 str. EDL933 (EHEC)	Z2338	0	3	0	1	2	3	0	1+1*	1	4	3	4	1	3	2	1+2*	1+1*	1+1*	1+1*	1+1*
nleG6-3	*E. coli* O157:H7 str. EDL933 (EHEC)	Z3921	0	0	1	1	0	0	0	0	1*	0	0	0	1	0	0	0	0	0	0	0
nleG7	*E. coli* O157:H7 str. EDL933 (EHEC)	Z2077	0	0	1	1	0	1	1	0	0	0	0	0	1	0	0	0	0	0	0	0
nleG8-2	*E. coli* O157:H7 str. EDL933 (EHEC)	Z3919	0	0	1	1	0	0	0	0	0	0	0	0	0	0	0	0	0	0	0	0
nleG-1	*E. coli* O55:H7 str. CB9615 (EPEC)	G2583_ RS01745	2	2	2	0	1	3	2+1*	3	3	2	1	2	2	4	4	3	3	3	3	3
nleG-2	*E. coli* O55:H7 str. CB9615 (EPEC)	G2583_ RS01740	0	0	0	0	0	1	0	1	1	0	1	2	1	1	1	0	1	1	1	1
nleH1	*E. coli* O157:H7 str. EDL933 (EHEC)	Z0989	1	1	1*	1*	2	3	2*	2+1*	2	0	1	2	2	1	2	1+2*	2	2+1*	1+2*	2
nleH2	*E. coli* O157:H7 str. EDL933 (EHEC)	Z6021	0	0	1*	0	0	0	1*	0	0	0	0	0	0	0	0	0	0	0	0	0
tccP/espF(U)	*E. coli* O103:H2 str. 12009 (EHEC)	ECO103_ RS12465	1	1	1	1*	1	1	1	1*	1	1	0	1	0	1	1	0	1	1*	1*	1
tir	*E. coli* O157:H7 str. EDL933 (EHEC)	Z5112	1	1	1	1	1	1	1	1	1	1	1	1	1	1	1*	1*	1	1	1*	1

*: pseudo gene; presence of mutations
0: Not detected
Numbers equal to or great than 1: the number of the homologs

Table S2. Detection of toxin-related genes in *E. albertii*.

Gene	Function	Clinical Strains									Chick strain	Wild bird strains									
		05-3106	07-3866	54-2045	2010C-3449	2011C-4180	2012EL-1823B	2013C-4143	2014C-4015	2014EL-1348	2014C-4356	RM9973	RM9974	RM9976	RM10507	RM10705	RM15112	RM15113	RM15114	RM15115	RM15116
eatA	Serine protease autotransporter toxin EatA	0	0	0	0	0	0	0	0	0	0	0	0	0	0	0	0	0	0	0	0
espC	Serine protease autotransporter toxin EspC	1	0	1*	0	0	0	0	0	0	0	0	0	0	0	0	0	0	0	0	0
espI	Serine protease autotransporter EspI	0	0	1	1	1*	0	1*	0	0	0	0	0	0	0	0	0	0	0	0	0
espP	Serine protease autotransporter EspP	0	0	0	0	0	0	0	0	0	0	0	0	0	0	0	0	0	0	0	0
pet	Serine protease autotransporter toxin Pet	0	0	0	0	0	0	0	0	0	0	0	0	0	0	0	0	0	0	0	0
pic	Serine protease autotransporter toxin Pic	0	0	0	0	0	0	0	0	0	0	0	0	0	0	0	0	0	0	0	0
sat	Serine protease autotransporter toxin Sat	0	0	0	0	0	0	0	0	0	0	0	0	0	0	0	0	0	0	0	0
sigA	Serine protease autotransporter toxin SigA	0	0	0	0	0	0	1*	0	0	0	0	0	0	0	0	0	0	0	0	0
tsh	Temperature-sensitive protease autotransporter hemagglutinin Tsh	1	0	0	0	0	0	0	0	0	0	0	0	0	0	0	0	0	0	0	0
vat	Vacuolating autotransporter toxin Vat	0	1	0	1	0	2	0	2	2	2	1	1	1	1 + 1*	1 + 1*	2	1 + 1*	2	1 + 1*	1 + 1*
cdiA	Contact-dependent inhibition effector tRNA nuclease	0	1	0	1*	0	0	0	0	0	1	1	0	1*	1	0	0	0	0	0	0
cdiB	ShlB/FhaC/HecB family hemolysin secretion/activation protein	0	1	0	1	0	0	0	0	0	1	1	0	1	1	0	0	0	0	0	0
cdtA	Cytolethal distending toxin type IV subunit CdtA	1	1	1	1	2	3	1	2	2	1	2	2	2	2	2	2	2	2	2	2
cdtB	Cytolethal distending toxin type IV nuclease subunit CdtB	1	1	1*	1	1+1*	2+1*	1	2	2	1	2	2	2	2	2	2	2	2	2	2
cdtC	Cytolethal distending toxin type IV subunit CdtC	1	1	0	1	2	3	1	2	2	1	2	2	2	2	2	2	2	1+1*	2	2
cnf1	Cytotoxic necrotizing factor CNF1	0	0	0	0	0	0	0	0	0	0	0	0	0	0	0	0	0	0	0	0
eltA	Heat-labile enterotoxin LT subunit A	0	0	0	0	0	0	0	0	0	0	0	0	0	0	0	0	0	0	0	0
eltB	Heat-labile enterotoxin LT subunit B	0	0	0	0	0	0	0	0	0	0	0	0	0	0	0	0	0	0	0	0
sta1	Heat-stable enterotoxin ST-I group a	0	0	0	0	0	0	0	0	0	0	0	0	0	0	0	0	0	0	0	0
hylA	Enterohemolysin EhxA	0	0	0	0	0	0	0	0	0	0	0	0	0	0	0	0	0	0	0	0
hlyB	Enterohemolysin T1SS ABC transporter permease/ATPase EhxB	0	0	0	0	0	0	0	0	0	0	0	0	0	0	0	0	0	0	0	0
hlyC	Enterohemolysin-activating lysine-acyltransferase EhxC	0	0	0	0	0	0	0	0	0	0	0	0	0	0	0	0	0	0	0	0
hlyD	Enterohemolysin T1SS ABC transporter subunit EhxD	0	0	0	0	0	0	0	0	0	0	0	0	0	0	0	0	0	0	0	0
hlyE	Hemolysin HlyE	0	0	0	0	0	0	0	0	0	0	0	0	0	0	0	0	0	0	0	0
efa1	lymphostatin Efa1/LifA	0	0	0	1	0	1*	0	1*	1*	0	0	0	0	0	0	0	0	0	0	0
senB	Enterotoxin production-related protein TieB	0	0	0	0	0	0	0	0	0	0	0	0	0	0	0	0	0	0	0	0
toxB	Toxin B	1	1*	0	0	1*	1	0	1	1	1	1	1	1	1	1	1	1	1	1	1*
stx1	Shiga toxin 1	0	0	0	0	0	0	0	0	0	0	0	0	0	0	0	0	0	0	0	0
stx2	Shiga toxin 2	0	0	0	0	1	1	0	1	1	0	1	1	1	1	1	1	1	1	1	1
eae	Intimin	1	1	1	1	1	1	1	1	1	1	1	1	1	1	1	1	1	1	1	1

*: pseudo gene; presence of mutations
0: Not detected
Numbers equal to or great than 1: the number of the homologs

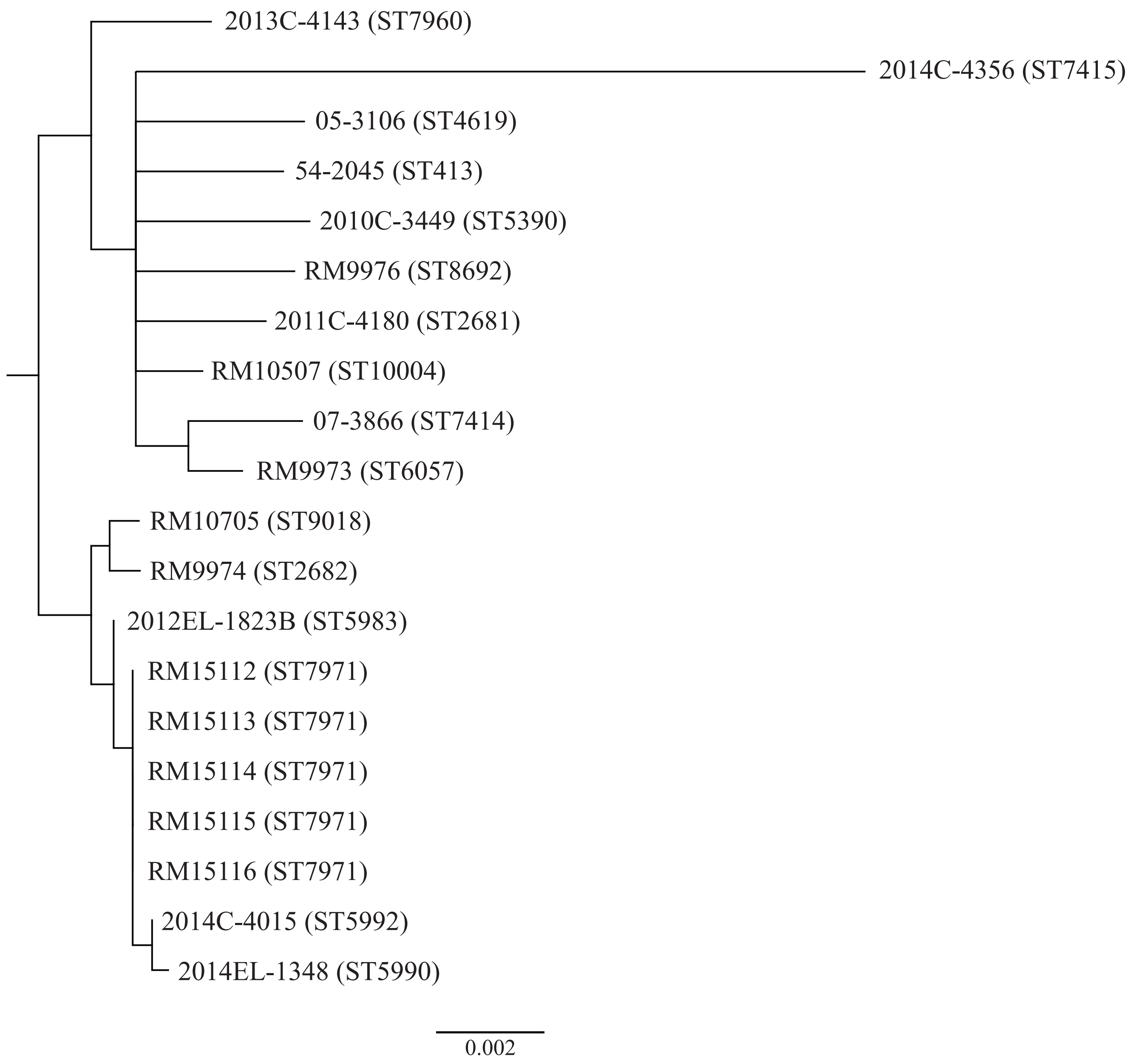

Figure S1. Phylogeny of *E. albertii* strains based on seqeunces of the MLST loci. The concatenated sequences of the seven loci (*adk, fumC, gyrB, icd, mdh, purA, and recA*) were aligned using Clustal Omega and the consensus sequence was used to construct phylogeny tree in Geneious Prime® as detailed in Materials and Methods. The sequence type (ST) was determined using MLST 2.0 service at the Center for Genomic Epidemiology with the *Escherichia coli* #1 configuration.

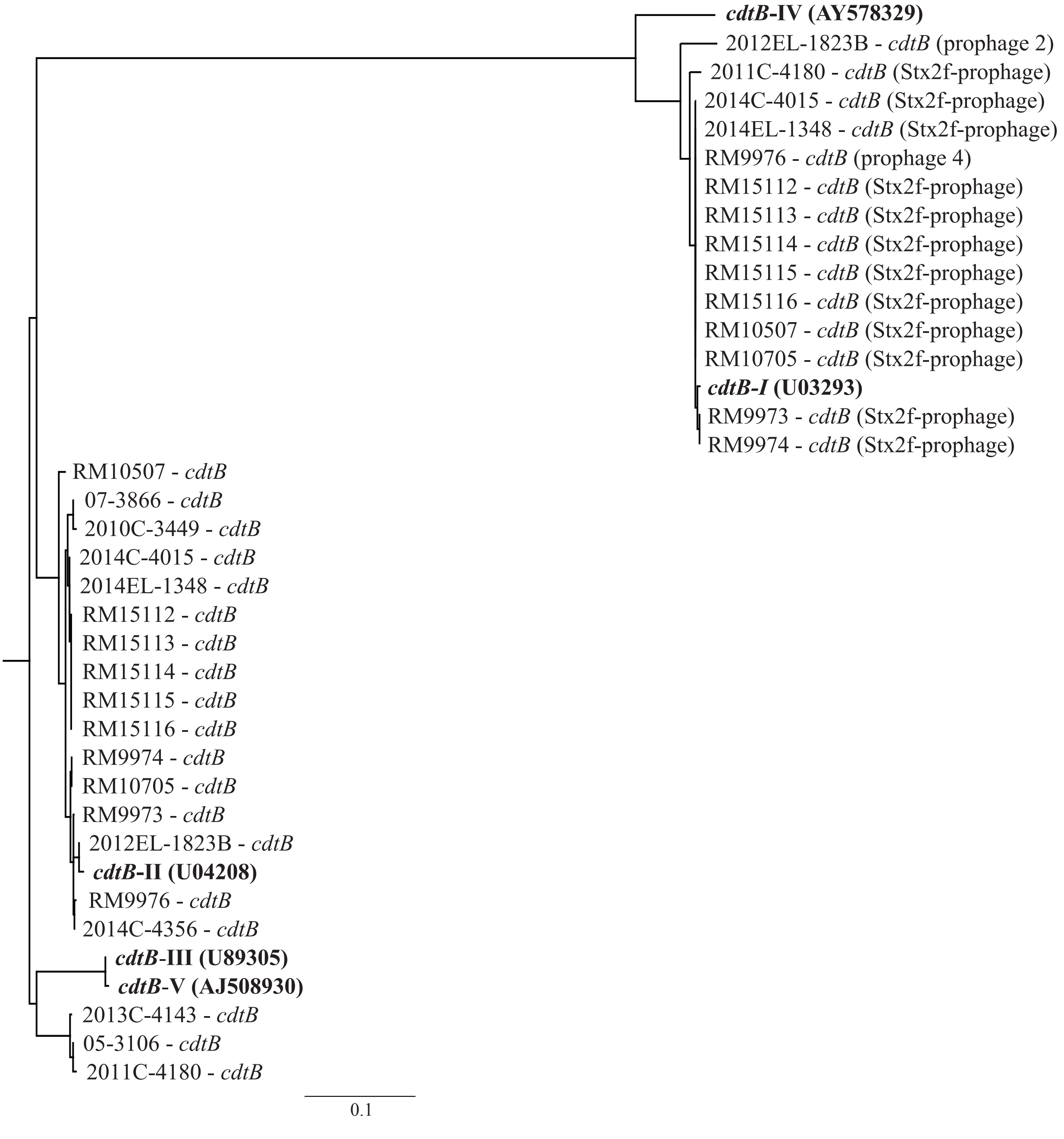

Figure S2. Phylogeny of *E. albertii cdtB*. Boldface indicates reference genes for five subtypes. The GenBank accession numbers are shown in parentheses. The *cdtB* gene located on the Stx2f-prophage in strain 2012EL-1823 was not included in the analyses due to an large insertional mutation in the *cdtB* gene (Loucs tag, AYN53_RS14910).
